# A Comparative Study of Intrathecal Fentanyl and Nalbuphine as an Adjuvant to Hyperbaric Bupivacaine for Spinal Anesthesia in Lower Limb Orthopedic Surgeries: A Prospective, Double-Blind, Randomized Controlled Study

**DOI:** 10.7759/cureus.41230

**Published:** 2023-06-30

**Authors:** Subhaprada Satapathy, Laba K Nayak, Sanjaya K Behera, Ganesh C Satapathy, Rasulata Swain, Saswati Das

**Affiliations:** 1 Anaesthesiology, Kalinga Institute of Medical Sciences, Bhubaneswar, IND

**Keywords:** orthopedic anesthesia, post operative pain, nalbuphine, fentanyl, spinal anaesthesia

## Abstract

Background: Spinal anaesthesia is the most commonly used technique for lower limb orthopaedic surgeries as it is economical and easy to administer. Opioids as adjuvants to local anaesthetics during spinal anaesthesia have played a vital role in reducing post-operative pain qualitatively and effectively.

Methods: This prospective randomised study was conducted on 100 patients divided into two groups scheduled for lower limb orthopaedic surgeries. Group bupivacaine fentanyl (BF) received 25 mcg of fentanyl with 15 mg of bupivacaine and Group bupivacaine nalbuphine (BN) received 1 mg of nalbuphine and 15 mg of 0.5% bupivacaine. The aim of the study was to compare the analgesic efficacy of intrathecal fentanyl and nalbuphine as an adjuvant to hyperbaric bupivacaine for spinal anaesthesia. Duration of effective analgesia, haemodynamic parameters, onset and duration of sensory and motor block, adverse effects, and visual analogue scale (VAS) score were assessed.

Results: Duration of effective analgesia was 388±24.88 minutes in the BN group and was higher (p-value <0.001) in comparison to the BF group, which was 304.70±15.76 minutes.

Conclusion: Nalbuphine was more effective than fentanyl in providing post-operative analgesia when used as an adjuvant to hyperbaric bupivacaine.

## Introduction

Intrathecal adjuvants to local anaesthesia have been introduced to boost clinical effectiveness and duration of analgesia following orthopaedic surgical operations as spinal anaesthesia alone provides poor postoperative analgesia. Intrathecal opioid effectively extends postoperative analgesia [1,2]. Opioid analgesics cause the main afferent neuron’s opioid receptors to become active, which in turn cause pain-modulating systems to become active. Inhibiting the production of excitatory neurotransmitters or directly reducing neurotransmission are both possible effects of their activation [3,4]. Fentanyl, an opioid agonist acts on mu receptors, causing supraspinal and spinal analgesia as well as drowsiness, nausea, vomiting, pruritus, and respiratory depression. Nalbuphine, an opioid agonist-antagonist primarily affecting kappa in the substantia gelatinosa of the spinal cord, has been shown to improve the quality of perioperative analgesia without the side effects of pure agonists [5].

Only a few trials have studied the efficacy of intrathecal fentanyl and nalbuphine as adjuvants to hyperbaric bupivacaine in lower limb orthopaedic surgeries [6,7]. The current study aims to examine the analgesic effect and duration of analgesia provided by intrathecal nalbuphine and fentanyl as adjuvants to hyperbaric bupivacaine in patients undergoing lower limb orthopaedic procedures.

## Materials and methods

The study was a randomized, prospective, double-blind study done in the Department of Anesthesiology at Pradyumna Bal Memorial Hospital, Kalinga Institute of Medical Sciences, Bhubaneswar, India. The Institutional Ethics Committee of Kalinga Institutional Medical Sciences approved the study (approval number: KIMS/KIIT/IEC/408/2020). This study was registered with the Clinical Trial Registry of India (CTRI/ 2021/01/030252). All participants were asked to sign an informed consent form after being educated about spinal anaesthesia and the risks involved. A total of 100 suitable cases were included in our study.

Inclusion and exclusion criteria

Inclusion criteria were patients posted for orthopaedic procedures on the lower limbs, American Society of Anesthesiologists (ASA) I and II, and age of 18-65 years. Exclusion criteria were: body mass index (BMI) more than 30, height under 155 cm, contraindications to spinal anaesthesia (patient refusal, increased intracranial tension, puncture site infection, coagulation disorder, hemodynamic instability, spinal deformities), patients using tranquillizers, antipsychotics, hypnotics, or other central nervous system depressants, patients who have previously experienced adverse reactions to opioid reactions, and pregnant patients.

Procedure

Each patient underwent a full pre-anaesthetic evaluation, which included a review of their medical history, clinical examination, systemic evaluation, and spine examination. Patients were instructed on how to use a visual analogue scale (VAS). Alprazolam 0.5 mg was given the night before surgery and on the morning of the operation with a sip of water.

A computer-generated randomization table was used to assign the 100 patients at random to two groups (50 each) and sequentially numbered opaque sealed envelopes were used for concealment. Group bupivacaine fentanyl (BF) received 25 mcg (0.5 ml) of fentanyl together and 15 mg of diluted 0.5% hyperbaric bupivacaine, and Group bupivacaine nalbuphine (BN) received 1 mg (0.5 ml) of nalbuphine and 15 mg of 0.5% hyperbaric bupivacaine.

Ringer lactate or regular saline at a rate of 15 ml/kg was used to preload the patient. Standard monitoring included a pulse oximeter, non-invasive blood pressure (NIBP), and electrocardiogram. Under all aseptic conditions, a 25G Quincke spinal needle was softly inserted in the midline of the L3-L4 /L4-L5 intervertebral space and advanced to the subarachnoid space. The unimpeded flow of cerebrospinal fluid via the needle verified the needle's location in the subarachnoid area. With the bevel end cephalad, the study medication (3.5 ml) was slowly injected into the subarachnoid area at a rate of 0.25 ml/sec. The patient was immediately placed in a supine position once the needle was withdrawn. A face mask delivering 100% oxygen at a flow rate of 4 L/minute was used. Dermatomes levels were checked every two minutes until the level of T10 was reached. Loss of cold sensation (cotton swab soaked in spirit) in the mid-axillary line and loss of pinprick sensation to a 23G hypodermic needle were used to determine sensory level. Surgery was commenced after T10 level was reached. A modified Bromage scale was used to evaluate the motor block that immediately followed the sensory block.

Following surgery the sensory and motor status were checked every two minutes for the first 10 minutes, then every five minutes for the next 10 minutes, and finally every 15 minutes until the sensory level regressed to dermatome S2 and the motor scale reached modified Bromage grade 0.

The following variables were defined [6]: (i) Time from the end of the study medication injection until the patient no longer feels the pinprick at T10 level is referred to as the time taken for sensory blockade to reach T10; (ii) Term time taken for maximal sensory blockade refers to the amount of time it took after the study drug injection to reach the highest level of sensory blockade to level T10; (iii) Term time taken for maximum motor blockade refers to the period of time between the end of the study medication injection and the onset of the maximum motor blockade; (iv) Duration of sensory blockade refers to the period of time from the time the study drug is injected and when the patient first perceives sensation at the S2 dermatome; (v) Duration of motor blockade is defined as the time needed for modified Bromage scale regression from grade 3 to grade 0; and (vi) Interval between achieving the maximal level of sensory blockade and the need for the first rescue dose of analgesia is defined as the duration of effective analgesia.

The modified Bromage scale was used to subjectively rate the degree of motor blockage in the lower limbs [6]: 1 = Patient is able to move the hip, knee, or ankle; 2 = The patient can move the knee and ankle but not the hip; 3 = The patient can move their ankle but not the hip or knee; 4 = The patient cannot move the hip, knee, or ankle; 5 = Total paralysis.

Adverse effects like nausea, vomiting, bradycardia, hypotension, pruritus, sedation, and respiratory depression were noted both during and following surgery and were treated as per institutional protocol. Sedation was scored on the Ramsay sedation scale [6].

Following surgery, patients were moved to the post anaesthesia care unit after measuring pulse, blood pressure, oxygen saturation, respiration rate, and clinical evaluation of motor as well as sensory blockade. Continuous monitoring and recordings at regular intervals were done until the complete return of sensory and motor function.

Using a VAS with a range of 0 to 10 (with 0 denoting no pain and 10 denoting the most intense pain), the postoperative pain score was recorded. Injection paracetamol (15 mg/kg) was administered as rescue analgesia when VAS was > 4.

Data analysis

Considering the mean difference and effect size of a previous study by Umesh et al. [6] with 95% power and 5% alpha error, 50 patients were required in each study arm. Data were collected, tabulated, and statistically analyzed using IBM SPSS Statistics for Windows, Version 25.0 (Released 2017; IBM Corp., Armonk, New York, United States). Quantitative data were expressed as mean ± SD. Qualitative data were expressed as numbers and percentages. A probability value (P-value) < 0.05 was considered statistically significant. Chi‑square/fisher exact test was used to check the association between two categorical variables. Student’s t-test was used to test the significance in difference between the two groups.

## Results

**Figure 1:**
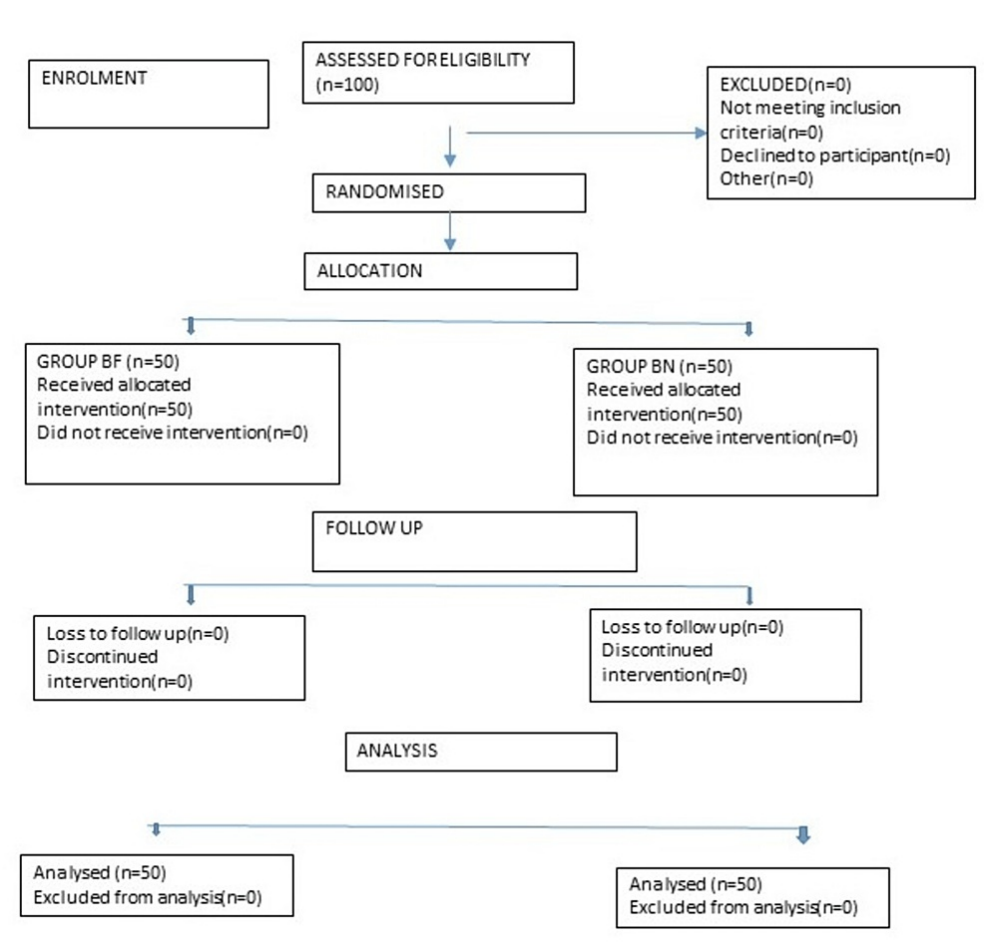
CONSORT diagram CONSORT: Consolidated Standards of Reporting Trials; BF: Group bupivacaine fentanyl; BN: Group bupivacaine nalbuphine

The study was conducted with a total of 100 patients randomly divided into two groups of 50 each (Figure 1).

The demographic data such as age, sex, BMI, ASA, and duration of surgery were comparable in both groups (p>0.05) as shown in Table 1.

**Table 1:** Demographic characteristics ASA: American Society of Anesthesiologists; BN: bupivacaine nalbuphine; BF: bupivacaine fentanyl

Characteristics	Group BN (n=50)	Group BF (n=50)	p-value
Age (years), mean±SD	45±15	45±14	0.745
BMI (kg/m^2^), mean±SD	22.59±2.07	22.32±1.92	0.574
Sex, n (%)			
Male	34 (68%)	29 (58%)	
Female	16 (32%)	21 (42%)	
ASA, n (%)			
ASA I	32 (64%)	36 (72%)	
ASA II	18 (36%)	14 (28%)	
Duration of surgery (hours), mean±SD	1.84±0.26	1.73±0.27	0.198

The time taken to reach T10 segment is faster in the BN (p=0.822) group. The highest sensory level was achieved faster in the BF group (p=0.225) (Table 2). The time taken to reach the highest motor block was faster in the BF group than in the BN group (p=0.529) (Table 2). The mean time taken for sensory regression to S2 and motor block regression to Bromage 0 was significantly lesser in the BF group than in the BN group (p<0.001) (Table 2). The mean duration of effective analgesia was significantly longer in the BN group than in the BF group (p<0.001) (Table 2).

**Table 2:** Block characteristics BN: bupivacaine nalbuphine; BF: bupivacaine fentanyl

Block Characteristics (minutes)	Group BN (n=50), Mean ± SD	Group BF (n=50), Mean ± SD	p-value
Time taken to reach T10 segment	0.3±01.14	3.00±1.26	0.822
Onset of motor block	2.53±1.28	2.40±1.22	0.6888
Time to reach highest sensory block	8.27±2.91	7.40±2.58	0.225
Time to reach highest motor block	5.80±2.48	5.40±2.42	0.529
Regression to S2	283.90±9.27	269.90±7.66	<0.001
Duration of motor block	280±13.96	263±14.62	<0.001
Duration of effective analgesia	388.40±24.88	304.70±15.76	<0.001

From 60 to 720 minutes, we found that the mean VAS score in the BN group was significantly lower than in the BF group (Table 3). 

**Table 3:** VAS score at different time intervals from 60 to 720 minutes VAS: visual analogue scale; BN: Group bupivacaine nalbuphine; BF: Group bupivacaine fentanyl

VAS score	Group	Count	Mean	SD	Minimum	Maximum	p value
60	BF	50	1.44	0.54	0.00	2.00	<0.001
	BN	50	0.18	0.39	0.00	1.00	
120	BF	50	2.44	0.54	1.00	3.00	<0.001
	BN	50	1.20	0.40	1.00	2.00	
180	BF	50	3.44	0.54	2.00	4.00	<0.001
	BN	50	2.20	0.40	2.00	3.00	
240	BF	50	2.16	2.01	0.00	4.00	<0.001
	BN	50	3.20	0.40	3.00	4.00	
300	BF	50	0.08	0.27	0.00	1.00	<0.001
	BN	50	3.24	1.55	0.00	4.00	
360	BF	50	0.22	0.42	0.00	1.00	0.05
	BN	50	0.08	0.27	0.00	1.00	
420	BF	50	0.72	0.45	0.00	1.00	<0.001
	BN	50	0.16	0.37	0.00	1.00	
480	BF	50	1.06	0.37	0.00	2.00	0.003
	BN	50	0.76	0.52	0.00	2.00	
540	BF	50	1.48	0.50	1.00	2.00	<0.001
	BN	50	1.12	0.33	1.00	2.00	
600	BF	50	1.98	0.14	1.00	2.00	<0.001
	BN	50	1.56	0.50	1.00	2.00	
660	BF	50	2.46	0.50	2.00	3.00	<0.001
	BN	50	1.96	0.35	1.00	3.00	
720	BF	50	2.92	0.27	2.00	3.00	<0.001
	BN	50	2.48	0.50	2.00	3.00	

Fortunately, in this study, patients did not face any serious adverse events in either group (p>0.05) (Table 4). Although heart rate was higher in the BF group, it was not statistically significant (p>0.05) (Table 5). Mean arterial pressure was comparable in both groups (p>0.05) (Table 6). 

**Table 4:** Side effects of the study participants observed in the two groups BN: bupivacaine nalbuphine; BF: bupivacaine fentanyl

Adverse effects	Group BF (n=50)	Group BN (n=50)	p-value
Vomiting	3	2	0.646
Hypotension	10	5	0.161
Pruritus	2	0	0.153
Sedation	0	0	1.000
Bradycardia	4	3	0.695
Respiratory depression	0	0	1.000

**Table 5:** Heart rate of the study population in the two groups at different time intervals BN: Group bupivacaine nalbuphine; BF: Group bupivacaine fentanyl

Heart rate at different minutes	Groups	Count	Mean	SD	Minimum	Maximum	p-value
At 0 minutes	BF	50	73.96	9.37	58.00	96.00	0.257
	BN	50	71.56	10.76	58.00	96.00	
At 15 minutes	BF	50	74.80	9.77	52.00	100.00	0.144
	BN	50	72.90	10.87	52.00	99.00	
At 30 minutes	BF	50	76.36	9.80	59.00	98.00	0.614
	BN	50	73.78	11.28	58.00	98.00	
At 45 minutes	BF	50	77.80	10.31	63.00	102.00	0.137
	BN	50	75.24	11.06	56.00	102.00	
At 60 minutes	BF	50	77.80	11.57	58.00	110.00	0.143
	BN	50	77.16	12.01	52.00	102.00	
At 90 minutes	BF	50	76.53	12.28	56.00	112.00	0.602
	BN	50	73.90	10.78	54.00	104.00	
At 120 minutes	BF	50	77.50	6.66	59.00	92.00	0.192
	BN	50	76.35	9.44	52.00	92.00	

**Table 6:** The mean arterial pressure measured in the two groups at different time intervals BN: Group bupivacaine nalbuphine; BF: Group bupivacaine fentanyl

Mean arterial pressure at different times	Group	Count	Mean	SD	Minimum	Maximum	p-value
At 0 minutes	BF	50	85.50	6.41	74.00	104.00	0.516
	BN	50	86.60	6.63	73.00	100.00	
At 15 minutes	BF	50	86.84	6.33	79.00	103.00	0.134
	BN	50	86.98	7.41	76.00	103.00	
At 30 minutes	BF	50	86.52	6.85	72.00	106.00	0.364
	BN	50	86.08	6.45	73.00	98.00	
At 45 minutes	BF	50	86.60	6.73	72.00	99.00	0.581
	BN	50	87.22	6.39	72.00	100.00	
At 60 minutes	BF	50	86.42	7.71	74.00	104.00	0.133
	BN	50	88.66	7.73	74.00	102.00	
At 90 minutes	BF	50	87.51	6.09	72.00	98.00	0.618
	BN	50	88.34	6.72	77.00	107.00	
At 120 minutes	BF	50	87.40	6.45	74.00	102.00	0.326
	BN	50	90.41	7.36	74.00	102.00	

## Discussion

For lower limb procedures, single-shot subarachnoid blocks are a frequent anaesthetic strategy. To offer postoperative pain relief and extend the duration while reducing the dose of local anaesthetic, intrathecal adjuvants are utilised with local anaesthetics. Bupivacaine when used alone for subarachnoid block, lasts for a short time and the duration of postoperative analgesia is also reduced. Opioids are frequently used in conjunction with local anaesthetics to increase hemodynamic stability while increasing the duration of motor and sensory block; 2.5 mg nalbuphine is equipotent to fentanyl 25 μg [8]. After an expansive search of the literature (Pubmed and Medline), a limited study was found to have compared 1 mg nalbuphine with 25 μg fentanyl as intrathecal adjuvants [9]. 

Nalbuphine when used as an intrathecal adjuvant, significantly delayed the onset of sensory block compared to patients receiving intrathecal fentanyl in our study. However, Shakooh et al. and Ahluwalia et al. reported earlier onset in patients getting nalbuphine intrathecally as compared to the control group [10,11]. Thote et al. reported no difference in the onset of sensory block [12]. Our study is supported by Manjula et al. [13] and Sharma et al. [9], who concluded that nalbuphine greatly delayed the onset of sensory block as compared to fentanyl.

In the current study, patients receiving intrathecal fentanyl experienced faster onset of motor block than patients receiving intrathecal nalbuphine. According to Sharma et al., patients receiving intrathecal fentanyl experienced complete motor block substantially sooner than patients receiving intrathecal nalbuphine, despite both being equivalent to control [9]. The complete motor block is defined as the duration to attain Bromage 4. Intrathecal fentanyl appears to produce a complete motor block much more quickly than nalbuphine, according to research by Gomaa et al. [14].

Our results showed similar findings to that of Mukherjee et al, who observed longer sensory block in all the groups receiving nalbuphine, as compared to the control [15]. In the indexed study by Gomaa et al, the duration of sensory block was found to be longer in the fentanyl group as compared to patients receiving nalbuphine {14].

Patients who got fentanyl had significantly higher VAS scores than those who received nalbuphine at all time intervals from 60 to 720 minutes. Duration of effective analgesia was determined from the point of subarachnoid block to the administration of the first rescue analgesic dose at VAS score > 4. When compared, the effective analgesia was significantly longer in patients receiving intrathecal nalbuphine (323.18±57.39 minutes) than in intrathecal fentanyl (287.05±78.87 minutes). Our study findings are consistent with those of other studies, which reported a considerable lengthening of the duration of spinal analgesia with nalbuphine [13-15].

The hemodynamics in the current investigation was discovered to be comparable across the two groups, which was alike to the findings of Sharma et al. [9]. In a study by Sapate et al., increased mean heart rate, systolic blood pressure, and diastolic blood pressure were seen in cases who received nalbuphine correlated to the control group, indicating a substantial difference in hemodynamic profile [16].

Fortunately, neither group of patients in this trial experienced any significant negative outcomes. However, the incidence of headache, nausea, and vomiting in all the groups was comparable to the study by Sharma et al. [9]. Gomaa et al. [14] showed that the incidence of side effects with nalbuphine did not significantly increase. Ahluwalia et al. reported a higher incidence of nausea and vomiting in the nalbuphine group [11]. Mukherjee et al. found that patients getting 0.8 mg of nalbuphine experienced more hypotension, nausea, vomiting, itching, and bradycardia than those receiving 0.2 mg and 0.4 mg of the drug [15]. Patients receiving intrathecal nalbuphine experienced a considerably higher incidence of drowsiness, according to studies by Shakooh et al. [10] and Thote et al. [12]. To confirm the side-effect profile of intrathecal nalbuphine, additional research may be needed.

The limited sample size would be the study's main drawback. Additional research must be done on a wider population of patients as well as those undergoing different surgical procedures. 

## Conclusions

Opioids as adjuvants to intrathecal bupivacaine is a commonly used intervention to achieve good postoperative outcomes. We conclude that both fentanyl and nalbuphine were equally efficacious in providing excellent intraoperative surgical anaesthesia and postoperative analgesia with good hemodynamic stability. The fentanyl group had a faster onset of both sensory and motor blockade as compared to the nalbuphine group, though it was not statistically significant. The duration of effective analgesia was, however, significantly more in the nalbuphine group.
